# Quantitative Analysis of Polyphenols in *Lonicera caerulea* Based on Mid-Infrared Spectroscopy and Hybrid Variable Selection

**DOI:** 10.3390/molecules31040750

**Published:** 2026-02-23

**Authors:** Haiwei Wu, Xuexin Li, Jianwei Liu, Zhihao Wang, Yuchun Liu

**Affiliations:** College of Engineering and Technology, Jilin Agricultural University, Changchun 130118, China; haiwei@jlau.edu.cn (H.W.); ljw1101@mails.jlau.edu.cn (J.L.); zhihaow@mails.jlau.edu.cn (Z.W.); 20230286@mails.jlau.edu.cn (Y.L.)

**Keywords:** *Lonicera caerulea*, polyphenol, mid-infrared spectroscopy, variable selection, high-dimensional small-sample, XGBoost

## Abstract

*Lonicera caerulea* L. (blue honeysuckle) is rich in antioxidant polyphenols, and rapid and accurate determination of its polyphenol content is of great significance for functional food quality control. This study proposed a hybrid variable selection strategy designed for high-dimensional small-sample scenarios and developed a quantitative prediction model for polyphenol content based on mid-infrared (MIR) spectroscopy. A total of 191 *Lonicera caerulea* samples were collected from Northeast China, and 7468-dimensional spectral data were acquired using a Fourier transform infrared spectrometer. Polyphenol reference values were determined by the Folin–Ciocalteu method. Samples were divided into calibration (*n* = 152) and prediction (*n* = 39) sets using the SPXY algorithm. Among the 10 preprocessing methods evaluated, MSC combined with Savitzky–Golay first derivative achieved the best performance and was therefore used for subsequent modeling. The proposed hybrid variable selection method (VIP1.0∩RFR30%) intersected PLS variable importance in projection (VIP ≥ 1.0) with the top 30% important variables from random forest regression, selecting 984 key wavelengths and achieving 86.8% dimensionality reduction. A three-stage hyperparameter tuning strategy was implemented across four models (PLS, RFR, SVR, and XGBoost) to validate feature stability and control overfitting. The optimized XGBoost model achieved excellent performance on the independent test set ($R^{2}$ = 0.92, RMSE = 0.098, RPD = 3.47). Compared with the classical CARS method ($R^{2}$ = 0.78, RPD = 2.14), $R^{2}$ improved by 16.3% and RPD improved by 55.2%. The results demonstrate that the proposed hybrid variable selection strategy can effectively address the challenges of high-dimensional MIR spectral data in small-sample modeling, providing a reliable tool for rapid and non-destructive quantitative analysis of polyphenols in *Lonicera caerulea*.

## 1. Introduction

*Lonicera caerulea* L., commonly known as blue honeysuckle or haskap berry, is emerging as a high-value functional fruit in the global market due to its robust cold resistance and exceptional nutritional profile [1]. Recent pharmacological studies have confirmed that this berry is a rich source of polyphenolic compounds, particularly cyanidin-3-O-glucoside and chlorogenic acid, which exhibit potent antioxidant, anti-inflammatory, and hypoglycemic activities [2,3]. As the consumer demand for naturally derived functional ingredients grows, the food industry faces increasing pressure to strictly control the quality of *Lonicera caerulea* raw materials and processed products [4]. Consequently, developing rapid, eco-friendly, and accurate quantification methods for polyphenols is a priority for product standardization and adulteration detection [5].

Traditional chemical analysis methods, such as HPLC-MS and colorimetric assays, although accurate, are destructive, labor-intensive, and require hazardous organic solvents, making them unsuitable for real-time industrial monitoring [6]. In contrast, vibrational spectroscopy has gained prominence as a green analytical technology. While near-infrared (NIR) spectroscopy involves broad overtone bands, mid-infrared (MIR) spectroscopy (4000–400 cm^−1^) operates in the fundamental vibration region. This allows MIR to capture distinct “fingerprint” information of molecular functional groups—such as O-H stretching in phenols and C=C vibrations in aromatic rings—resulting in higher spectral specificity and clearer chemical interpretability for complex polyphenolic matrices [7,8]. However, raw MIR spectra typically contain thousands of wavenumbers with severe multicollinearity and noise, creating a high-dimensional feature space that poses significant challenges for modeling [9].

A critical bottleneck in agricultural spectral analysis is the “high-dimensional small-sample” problem (*p* >> *n*), where the number of spectral variables far exceeds the number of available biological samples [10]. In such scenarios, full-spectrum modeling is prone to overfitting and poor generalization, as irrelevant variables (noise) may mask the useful information related to the target analytes [11]. Recent studies in 2023 and 2024 have highlighted that aggressive feature selection is indispensable for improving model robustness and reducing computational complexity [12,13].

To address this, various variable selection algorithms have been developed, including Competitive Adaptive Reweighted Sampling (CARS), Successive Projections Algorithm (SPA), and Iterative Variable Subset Combination (IVSO) [14,15]. Although these single-criterion methods have shown success, they exhibit inherent limitations when applied to small-sample data. For instance, CARS is stochastic and may converge to local optima depending on the random split of calibration sets, while projection-based methods like PLS-VIP may select collinear variables that do not contribute to prediction accuracy [16]. Consequently, recent chemometric trends have shifted towards “hybrid” or “ensemble” strategies that combine the strengths of linear and nonlinear evaluation metrics to ensure the stability of selected feature subsets [17,18].

Therefore, this study proposes a novel hybrid variable selection strategy combining Partial Least Squares-Variable Importance in Projection (PLS-VIP) with Random Forest Regression (RFR) importance. This approach aims to address the instability of feature selection in high-dimensional small-sample MIR data of *Lonicera caerulea*. By integrating the global linearity assessment of VIP with the nonlinear feature ranking of RFR, and implementing a three-stage hyperparameter tuning framework, we aim to extract the most representative spectral fingerprints for polyphenol quantification.

The specific objectives of this study were to:

1. Evaluate different spectral preprocessing methods for MIR spectra of *Lonicera caerulea*;
2. Develop and validate a hybrid variable selection strategy (VIP1.0∩RFR30%) for high-dimensional small-sample data;
3. Compare the proposed method with classical CARS and single-criterion selection approaches;
4. Validate the universality of the selected variables across multiple machine learning models;
5. Establish an optimized prediction model for polyphenol content with high accuracy and robustness.

## 2. Results and Discussion

### 2.1. Spectral Preprocessing Optimization

The selection of an appropriate preprocessing method is fundamental to the success of spectroscopic modeling, as it directly affects the signal-to-noise ratio and the extraction of chemically relevant information. As shown in Table 1, the combination of MSC with SG first derivative (MSC + SG 1st Der) achieved the best performance among all evaluated methods, with *R*^2^ = 0.8735 and RPD = 2.8121, marginally outperforming SNV + SG first derivative (*R*^2^ = 0.8735, RPD = 2.8120).

**Figure 1:**
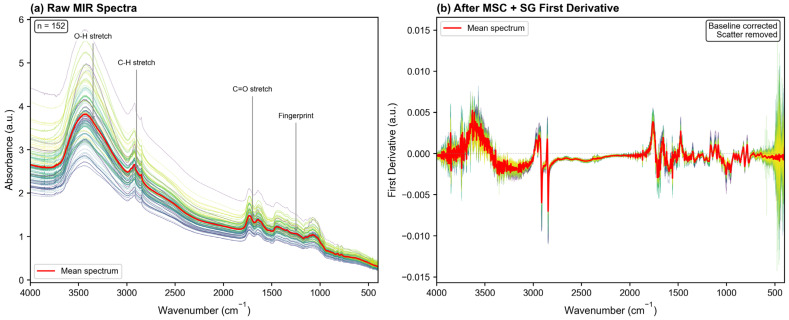
Mid-infrared spectra of *Lonicera caerulea* samples. (**a**) Raw MIR spectra of 152 calibration samples showing the spectral range from 4000 to 400 cm^−1^. Key absorption regions are annotated: O-H stretching (3200–3500 cm^−1^), C-H stretching (2800–3000 cm^−1^), C=O stretching (1600–1800 cm^−1^), and fingerprint region (1000–1500 cm^−1^). (**b**) Preprocessed spectra after multiplicative scatter correction (MSC) combined with Savitzky–Golay first derivative (window = 11, polynomial order = 2), showing improved baseline correction and enhanced spectral features.

The superior performance of MSC combined with SG first derivative can be attributed to the synergistic correction of both physical scattering and spectral overlaps. Powdered samples inherently suffer from severe light scattering effects due to non-uniform particle sizes, which manifest as multiplicative baseline shifts. MSC effectively mitigates these physical artifacts by linearizing the spectra against a reference mean. Simultaneously, the SG first derivative eliminates additive baseline drift and resolves overlapping absorption bands, thereby enhancing the resolution of specific functional groups [19]. This synergy between scatter correction (MSC) and derivative enhancement (SG 1st Der) has been consistently observed in MIR spectroscopic studies of plant materials, where physical interference from sample heterogeneity often obscures subtle chemical information [20]. Consequently, this combination maximizes the signal-to-noise ratio specific to the target analytes, making it the optimal choice for all subsequent analyses. The mid-infrared spectra of *Lonicera caerulea* before and after optimal pretreatment are shown in Figure 1.

### 2.2. Variable Selection and Comparative Evaluation

**Figure 2:**
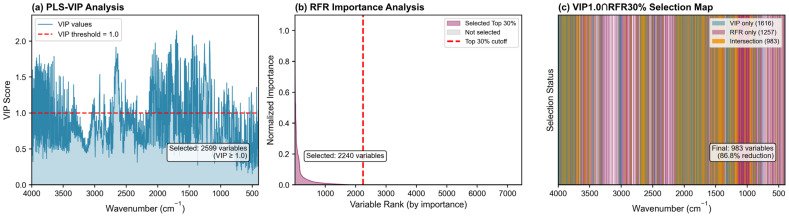
Hybrid variable selection strategy (VIP1.0∩RFR30%). (**a**) PLS-VIP profile showing variable importance in projection scores; wavelengths with VIP ≥ 1.0 (2599 variables) were selected as linearly important. (**b**) Random forest regression importance profile ranked by normalized Gini importance; the top 30% variables (2240 wavelengths) were selected as nonlinearly important. (**c**) Selection map showing the intersection of PLS-VIP and RFR selections, resulting in 984 characteristic wavelengths (86.8% dimensionality reduction) that are important from both linear and nonlinear perspectives.

Variable selection is critical for addressing the curse of dimensionality inherent in high-dimensional small-sample spectroscopic data. The results of the three-component variable selection approach are illustrated in Figure 2.

#### 2.2.1. PLS-VIP Analysis

PLS-VIP analysis identified 2599 wavelengths with VIP scores ≥ 1.0, representing 34.8% of the original 7468 variables (Figure 2a). These wavelengths are considered linearly important for polyphenol prediction from a chemometric perspective. The VIP profile reveals that high VIP regions are distributed across multiple spectral ranges (1100–1300, 1500–1800, and 2800–3500 cm^−1^), corresponding to various functional groups associated with polyphenolic compounds including phenolic hydroxyl groups, aromatic rings, and carbonyl moieties.

#### 2.2.2. RFR Importance Analysis

Complementing the linear PLS-VIP analysis, RFR importance analysis selected the top 30% of variables (2240 wavelengths) based on normalized Gini importance scores (Figure 2b). Notably, RFR identifies partially different important regions compared to PLS-VIP, reflecting the nonlinear relationships and complex interactions between spectral features and polyphenol content that linear methods may not fully capture. This observation aligns with previous findings that ensemble methods can reveal variable importance patterns distinct from those identified by linear approaches [21].

#### 2.2.3. Hybrid Selection (VIP1.0∩RFR30%)

The intersection of PLS-VIP ≥ 1.0 and RFR top 30% yielded 984 characteristic wavelengths (Figure 2c), representing a substantial 86.8% reduction in dimensionality from the original 7468 variables. These wavelengths satisfy dual importance criteria—significant from both linear and nonlinear perspectives—providing robust validation of their relevance to polyphenol prediction. The (Figure 2c) clearly illustrates the overlapping regions where both methods agree on variable importance.

#### 2.2.4. Comparative Evaluation and Mechanism Analysis

To rigorously evaluate the proposed hybrid strategy, the six candidate strategies constructed in Section 3.7.3 were compared using XGBoost 2.0.3 with fixed parameters to ensure a fair assessment. As shown in Table 2, the results reveal substantial performance differences driven by the varying selectivity of each strategy.

Performance Analysis: The proposed VIP1.0∩RFR30% strategy achieved the highest performance (*R*^2^ = 0.9097, RPD = 3.3270), significantly outperforming all other approaches.

Comparison within Candidate Pool: The Relaxed Linear Strategy (PLS-VIP-0.8) retained too many variables (4057), likely introducing collinear noise that degraded model performance (*R*^2^ = 0.8496). Conversely, the Strict Hybrid Strategy (VIP-1.2∩RFR-20%) was overly aggressive in elimination, resulting in the loss of useful spectral information (*R*^2^ = 0.8928). The “Balanced Hybrid” strategy successfully struck the optimal trade-off between noise reduction and information retention.

Comparison with CARS: The improvement over the classical CARS method was remarkable, with a 16.3% increase in *R*^2^ and a 55.2% increase in RPD. This result empirically validates the superiority of the intersection-based approach for this specific dataset.

Synergistic Mechanism of Superiority: The success of the hybrid strategy can be attributed to three synergistic mechanisms:

Filtering of False Positives via Dual Validation: Single-criterion methods often select “spurious variables”—wavelengths that appear statistically significant due to chance correlations in small datasets but lack chemical meaning. By requiring a variable to be validated by both a linear engine (PLS) and a nonlinear engine (RFR), the intersection approach acts as a “dual-lock” filter, effectively removing these false positives.

Complementary Feature Capture: PLS-VIP excels at identifying global linear trends associated with concentration changes, while RFR captures complex nonlinear spectral responses. The intersection retains variables that possess robustness across modeling paradigms, ensuring that selected features are not algorithm-specific artifacts.

Deterministic Stability: Unlike single-criterion methods that are sensitive to arbitrary thresholding, the hybrid approach benefits from the consensus of two independent importance measures, enhancing selection stability.

**Figure 3:**
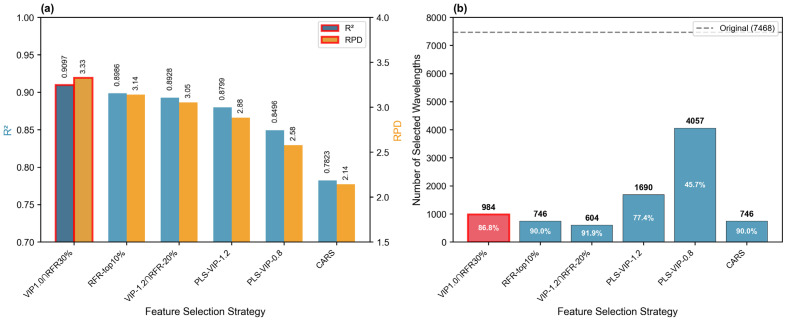
Comparison of six feature selection strategies. (**a**) Model performance (*R*^2^ and RPD) achieved by XGBoost using different variable subsets. (**b**) Number of selected wavelengths for each strategy. The proposed VIP1.0∩RFR30% strategy (highlighted in red) achieved the best performance while maintaining substantial dimensionality reduction.

Analysis of CARS Limitation: As illustrated in Figure 3, the relatively poor performance of CARS (*R*^2^ = 0.7823) in this study highlights the intrinsic limitations of iterative stochastic methods in high-dimensional small-sample scenarios (*p*/*n* ≈ 39). CARS relies on Monte Carlo sampling, which requires a sufficient sample density to statistically converge on the global optimum. When the sample size is limited relative to the number of variables, CARS is prone to becoming trapped in local optima or selecting unstable subsets due to its stochastic nature. This phenomenon was similarly observed by Yun et al. in their comparative study, which noted that simple importance-based selection can outperform iterative methods when sample size is the limiting factor.

### 2.3. Multi-Model Validation

A critical question in variable selection is whether the selected variables contain genuinely robust information or are merely optimized for a specific modeling algorithm. To address this concern, the VIP1.0∩RFR30% variable subset was validated across four machine learning models representing different algorithmic paradigms. As presented in Table 3, all models achieved satisfactory performance, demonstrating the universality of the selected variables.

**Figure 4:**
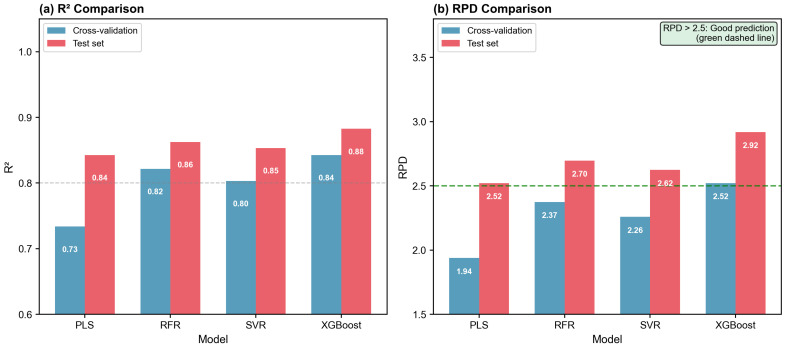
Multi-model validation results using VIP1.0∩RFR30% selected variables (984 wavelengths). (**a**) *R*^2^ comparison between cross-validation and test set for PLS, RFR, SVR, and XGBoost. (**b**) RPD comparison showing that all models achieved RPD > 2.5 on the test set, confirming the universality of the selected variables across different modeling paradigms.

Notably, all four models achieved RPD values greater than 2.5 on the independent test set, meeting the threshold for “good prediction capability” according to Williams’ criteria [22] (Figure 4). Although PLS and SVR showed slightly lower RPD values compared to XGBoost, their performance (RPD > 2.5) still qualifies as suitable for quantitative prediction applications. This cross-model consistency validates that the selected 984 wavelengths contain intrinsically robust information for polyphenol prediction, independent of the specific modeling algorithm employed.

XGBoost demonstrated the best overall performance (Test *R*^2^ = 0.8826, RPD = 2.9190), which can be attributed to its ability to capture complex nonlinear relationships through sequential gradient boosting while effectively controlling overfitting through built-in L1/L2 regularization [23,24]. The superior performance of ensemble methods (XGBoost, RFR) over linear PLS suggests that the relationship between MIR spectral features and polyphenol content involves nonlinear components that benefit from tree-based modeling approaches.

The universality demonstrated here is particularly important for practical applications, where different models may be preferred based on computational resources, interpretability requirements, or deployment constraints. The fact that even the simplest model (PLS) achieved acceptable performance (RPD = 2.5211) indicates that the selected variables capture fundamental spectral–chemical relationships rather than algorithm-specific patterns.

### 2.4. Optimized XGBoost Model Performance

Following the three-stage hyperparameter tuning strategy, the final XGBoost model was optimized exclusively on the VIP1.0∩RFR30% variable subset. As shown in Table 4, the optimized model achieved excellent prediction performance on the independent test set.

The optimized model parameters were: n_estimators = 100, max_depth = 3, learning_rate = 0.1, subsample = 0.78, colsample_bytree = 0.78, reg_alpha = 0.1, reg_lambda = 0.1. The relatively shallow tree depth (max_depth = 3) and moderate regularization parameters (reg_alpha = 0.1, reg_lambda = 0.1) reflect the conservative tuning approach designed to prevent overfitting in the small-sample scenario.

The prediction set *R*^2^ of 0.9168 and RPD of 3.4674 indicate “excellent prediction capability” according to Williams’ established criteria (RPD ≥ 3.0) [22]. Importantly, the relatively small gap between calibration *R*^2^ (0.9633) and prediction *R*^2^ (0.9168)—a difference of only 0.0465—suggests that overfitting was effectively controlled through the three-stage tuning strategy and regularization. This close alignment between training and test performance is particularly noteworthy given the high-dimensional small-sample nature of the data, where overfitting is a common concern.

**Figure 5:**
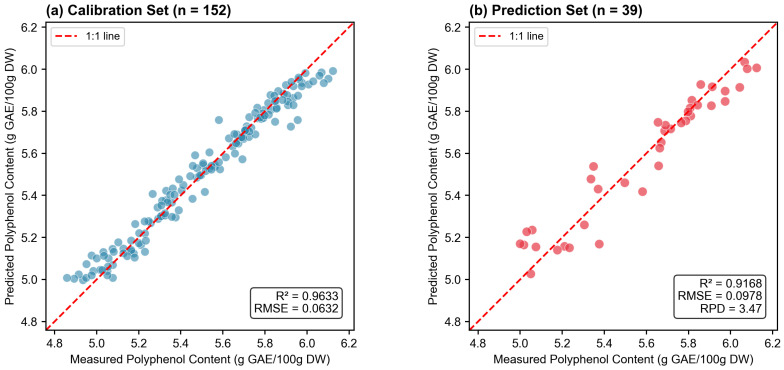
Scatter plots of predicted versus measured polyphenol content using the optimized XGBoost model with VIP1.0∩RFR30% selected variables. (**a**) Calibration set (*n* = 152) showing *R*^2^ = 0.9633 and RMSE = 0.0632. (**b**) Independent prediction set (*n* = 39) showing *R*^2^ = 0.9168, RMSE = 0.0978, and RPD = 3.4674, indicating excellent prediction capability. The dashed line represents the 1:1 reference line.

Figure 5 presents the scatter plot of predicted versus measured polyphenol content. The data points are closely distributed around the 1:1 reference line with minimal scatter, visually demonstrating the high accuracy and precision of the model. The absence of systematic bias (no curvature or offset from the reference line) further confirms the model’s reliability across the full range of polyphenol concentrations encountered in *Lonicera caerulea* samples.

### 2.5. Chemical Interpretation of Selected Wavelengths

**Figure 6:**
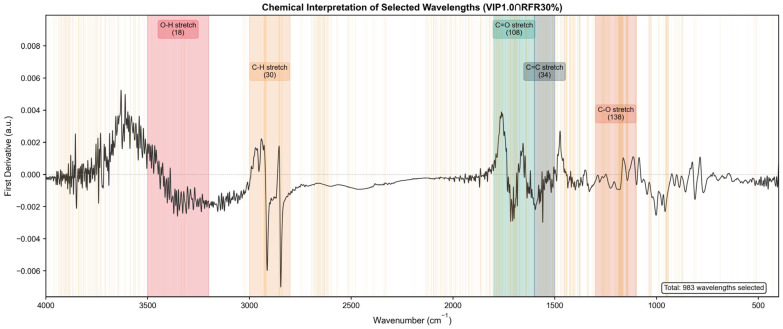
Chemical interpretation of selected wavelengths. The mean preprocessed spectrum is shown with highlighted regions corresponding to polyphenol-related functional groups: O-H stretching (3200–3500 cm^−1^, hydroxyl groups), C-H stretching (2800–3000 cm^−1^, aliphatic chains), C=O stretching (1600–1800 cm^−1^, carbonyl groups in flavonoids), C=C stretching (1500–1600 cm^−1^, aromatic rings), and C-O stretching (1100–1300 cm^−1^, phenolic hydroxyl groups). Orange vertical lines indicate the 984 wavelengths selected by VIP1.0∩RFR30%.

Beyond predictive performance, the chemical interpretability of selected variables is crucial for validating the spectral–chemical relationships underlying the model. Unlike “black-box” models, the 984 wavelengths selected by the VIP1.0∩RFR30% strategy are not randomly distributed; instead, they cluster in specific spectral regions that serve as the “molecular fingerprints” of the dominant polyphenols in *Lonicera caerulea* (e.g., cyanidin-3-O-glucoside and chlorogenic acid) (Table 5, Figure 6).

The Fingerprint Region (1000–1450 cm^−1^): A dense cluster of selected variables appears in the 1000–1300 cm^−1^ range. This region is dominated by C-O-C symmetric stretching and C-OH bending vibrations, which are chemically diagnostic of the glycosidic linkages and pyran rings found in anthocyanins. Since cyanidin-3-O-glucoside is the primary anthocyanin in haskap berries [25], the specific selection of bands at 1030 cm^−1^ and 1260 cm^−1^ strongly suggests that the model is directly detecting the glycosylated structure of the target analytes rather than relying on indirect correlations with sugar content.

The Aromatic Skeletal and Carbonyl Regions (1500–1750 cm^−1^): The hybrid strategy identified critical features corresponding to the core polyphenolic structure. The distinct peaks selected around 1515 cm^−1^ and 1600 cm^−1^ are assigned to aromatic ring C=C skeletal vibrations, providing direct evidence that the model successfully isolated the polyphenol backbone signal. Furthermore, variables selected in the 1680–1750 cm^−1^ range correspond to the C=O stretching of ester groups, characteristic of the ester bond connecting caffeic acid and quinic acid in chlorogenic acid [26]. By capturing these specific vibrations while excluding water interference regions (e.g., H-O-H bending near 1640 cm^−1^), the model effectively distinguishes the target analytes from the complex biological matrix.

Crucially, the hybrid strategy effectively excluded regions associated with strong water interference (e.g., broad O-H stretching >3000 cm^−1^ and H-O-H bending near 1640 cm^−1^), which are common sources of noise in fresh fruit analysis. The XGBoost feature importance analysis further corroborated this, ranking the aromatic C=C stretching (approx. 1520 cm^−1^) and conjugated C=O stretching (approx. 1650 cm^−1^) as the top contributors. This alignment between statistical importance and chemical theory validates that the VIP1.0∩RFR30% strategy is robust and mechanistically sound.

### 2.6. Comparison with Previous Studies

To contextualize the performance of the proposed method, Table 6 benchmarks the results against previous spectroscopic studies on berry polyphenol quantification. The comparison highlights a significant leap in prediction reliability.

The proposed method achieved superior performance compared to all previous studies, with improvements of 7.9–17.9% in *R*^2^ and 20.0–47.0% in RPD. This performance advantage stems from addressing specific limitations in prior methodologies:

Fundamental vs. Overtone Vibrations (MIR vs. NIR): Most previous studies [27,29] relied on Near-Infrared (NIR) spectroscopy. While NIR is non-destructive, its spectra consist of broad, overlapping overtones and combination bands that lack specificity. In contrast, this study utilized Mid-Infrared (MIR) spectroscopy to capture fundamental vibrations, providing sharper and more distinct spectral features (“fingerprints”) that allow for more accurate differentiation of polyphenols from other matrix components.

Stability in Variable Selection (Hybrid vs. CARS/SPA): The comparison with Ferrer-Gallego et al. [28] is particularly instructive. Although they also used MIR spectroscopy for polyphenols, their reliance on CARS resulted in a lower RPD (2.89 vs. 3.47). This discrepancy underscores the limitation of stochastic methods like CARS in small-sample scenarios, where they may converge to local optima. The proposed VIP∩RFR strategy ensures stability by enforcing dual validation (linear + nonlinear), leading to a more robust feature subset.

Modeling Non-linearity (XGBoost vs. PLS): Traditional PLS modeling assumes a linear relationship between spectra and concentration. However, in complex biological matrices, interactions between components often induce non-linear spectral responses. The use of XGBoost with regularization allowed the model to capture these subtle non-linear dependencies without overfitting, as evidenced by the high generalization capability on the test set.

Practical Significance: According to Williams’ criteria, an RPD between 2.5 and 3.0 (achieved by previous studies) allows for “good prediction,” but an RPD > 3.0 (achieved in this study, RPD = 3.47) marks a transition to “excellent prediction” suitable for process control. This suggests that the proposed methodology is not merely a screening tool but is sufficiently robust for routine quantitative quality control in the industrial processing of *Lonicera caerulea*.

Although this study focused on *Lonicera caerulea*, the proposed VIP∩RFR strategy addresses the fundamental “high-dimensional small-sample” challenge (*p* >> *n*) common in agricultural spectral analysis. The mechanism of using a “dual-lock” filter—requiring variables to be significant in both linear (PLS) and nonlinear (RFR) domains—is mathematically generic. Therefore, this strategy holds significant potential for transferability to other complex plant matrices (e.g., tea polyphenols, grape anthocyanins) where spectral overlapping and physical interference limit the effectiveness of single-criterion selection methods.

### 2.7. Practical Implications

The developed model has significant practical implications for the *Lonicera caerulea* industry and broader applications in functional food quality control:

- Rapid quality control: The MIR-based method enables polyphenol quantification within 2–3 min per sample (including sample loading and spectral acquisition), compared to 2–3 h required for traditional Folin–Ciocalteu colorimetric methods. This dramatic reduction in analysis time enables real-time quality monitoring during harvest and processing.
- Non-destructive analysis: Unlike chemical methods that consume samples, MIR-ATR analysis requires only ~20 mg of powder and leaves the sample intact for subsequent use or confirmatory testing. This is particularly valuable for precious samples or when multiple analyses are required.
- Cost-effective operation: After initial instrument investment (FTIR spectrometer with ATR accessory), the per-sample analysis cost is minimal—requiring no reagents, solvents, or consumables beyond routine maintenance. This makes the method economically viable for routine quality control applications.
- Standardization potential: The model can be deployed for quality grading and standardization of *Lonicera caerulea* products, supporting the development of industry standards for polyphenol content in functional foods. The excellent RPD (3.4674) indicates that the model is suitable for quality control applications where accurate quantification is required [22].
- Scalability for industrial deployment: The combination of rapid analysis, minimal sample preparation, and robust performance across different sample origins (three geographical regions) suggests strong potential for integration into production line quality control systems.

### 2.8. Limitations and Future Perspectives

While the proposed VIP1.0∩RFR30% strategy demonstrated excellent performance on the independent prediction set, we acknowledge certain limitations regarding model robustness. The variable selection was performed on the full calibration set (*n* = 152) prior to cross-validation. Although the prediction set (*n* = 39) was strictly isolated and never used during the feature selection or training phases, this “single-split” approach may still carry a risk of optimistic bias compared to nested cross-validation or multi-year external validation. The SPXY algorithm was employed to maximize the representativeness of the calibration set and mitigate sampling bias, yet the model’s stability across different harvest years or laboratories remains to be verified. Therefore, this study establishes the feasibility of the method, but future work utilizing large-scale external validation datasets is necessary before routine industrial deployment.

## 3. Materials and Methods

### 3.1. Sample Collection and Preparation

A total of 191 wild *Lonicera caerulea* samples were collected from three distinct geographical regions in Northeast China during the peak harvest season (July to August): Greater Khingan Mountains (65 samples), Lesser Khingan Mountains (63 samples), and Changbai Mountains (63 samples). Fresh berries were hand-picked at full maturity (uniform dark blue-purple skin color and softened flesh). Approximately 1.5 g of haskap pulp was collected for each sample, placed in sterile polyethylene bags, and immediately stored in portable refrigerators at 4 °C during transportation to the laboratory.

Upon arrival, samples were manually cleaned to remove leaves, twigs, and damaged fruits. To eliminate water interference in MIR spectra and stabilize the samples, berries were freeze-dried using a vacuum freeze dryer. The drying process lasted 48 h at −50 °C under 10 Pa pressure. The dried berries were then ground into fine powder using a high-speed universal grinder and passed through an 80-mesh sieve to ensure uniform particle size. The resulting powder was sealed in vacuum bags and stored at −20 °C in darkness until analysis to prevent polyphenol oxidation.

### 3.2. Reagents and Standards

The main reagents used for reference analysis included: Folin–Ciocalteu phenol reagent (Sigma-Aldrich, St. Louis, MO, USA); gallic acid standard (purity ≥ 98%, Sigma-Aldrich); anhydrous sodium carbonate (Na_2_CO_3_, analytical grade, Sinopharm Chemical Reagent Co., Ltd., Shanghai, China); ethanol (analytical grade, Fuyu Fine Chemical, Tianjin, China); and deionized water (18.2 MΩ·cm) generated by a Milli-Q system.

### 3.3. Reference Measurement: Total Polyphenol Content (TPC)

The TPC of haskap samples was determined using the Folin–Ciocalteu colorimetric method, which is the accepted industry standard.

Extraction Procedure: 0.5 g of freeze-dried haskap powder was accurately weighed and transferred to a centrifuge tube. 25 mL of 60% ethanol aqueous solution was added as the extraction solvent. The mixture was subjected to ultrasonic-assisted extraction (power 300 W, frequency 40 kHz) for 30 min at 40 °C. After extraction, the mixture was centrifuged at 5000 rpm for 10 min, and the supernatant was collected.

Colorimetric Reaction: 0.2 mL of the supernatant (diluted appropriately to fall within the linear range) was mixed with 1.0 mL of Folin–Ciocalteu reagent (diluted 1:10 with deionized water). The mixture was vortexed and allowed to react for 5 min. Subsequently, 1.0 mL of 7.5% (*w*/*v*) sodium carbonate solution was added to alkalize the medium. The final volume was adjusted to 10 mL with deionized water.

Standard Curve Preparation: A gallic acid stock solution (100 mg/L) was prepared by dissolving 10.0 mg of gallic acid standard in 60% ethanol and diluting to 100 mL in a volumetric flask. Working standard solutions at concentrations of 0, 1, 2, 4, 6, 8, and 10 mg/L were prepared by appropriate dilution of the stock solution with 60% ethanol. Each standard solution was subjected to the colorimetric reaction procedure described above, and absorbance was measured at 765 nm. The standard calibration curve was constructed by plotting absorbance (*A*) against gallic acid concentration (C, mg/L). The regression equation was:

(1) $$A=0.0098C+0.0012\;(R^{2}=0.9994)$$

The linear range was 0–10 mg/L with a detection limit of 0.05 mg/L.

Quantification: The mixture was incubated at room temperature (25 °C) for 60 min in the dark. The absorbance was measured at 765 nm using a UV-Vis spectrophotometer (Shimadzu UV-2600). The total polyphenol content was calculated using the following equation:

(2) $$TPC=\frac{\left(A-0.0012\right)\times V\times D}{\left(0.0098\times m\times 10\right)}$$

where TPC is the total polyphenol content (g GAE/100 g DW), A is the sample absorbance, V is the extraction volume (mL), D is the dilution factor, and m is the sample mass (g). The results were expressed as grams of gallic acid equivalents per 100 g of dry weight (g GAE/100 g DW). All measurements were performed in triplicate, and the mean value was used for modeling.

### 3.4. Mid-Infrared Spectral Acquisition

Spectral data were collected using a Fourier Transform Infrared Spectrometer (Thermo Scientific Nicolet iS50, Waltham, MA, USA) equipped with a high-sensitivity Deuterated Triglycine Sulfate (DTGS) detector and a single-bounce Diamond Attenuated Total Reflectance (ATR) accessory.

Laboratory Environmental Conditions: All spectral acquisitions were performed in a temperature- and humidity-controlled laboratory environment. The laboratory temperature was maintained at 25 ± 1 °C, and relative humidity was controlled at 45 ± 5%. The instrument was allowed to warm up and stabilize for at least 30 min before measurements to ensure stable operation of the light source and detector. The laboratory was equipped with an air purification system to minimize atmospheric CO_2_ and water vapor interference on the spectra. Samples were equilibrated in the laboratory environment for at least 15 min before measurement to eliminate temperature-induced spectral variations.

Measurement Protocol: The background spectrum of clean air was collected prior to each sample to subtract atmospheric interferences (CO_2_ and H_2_O vapor). Approximately 20 mg of haskap powder was placed directly onto the diamond crystal. A pressure clamp was applied to ensure uniform contact between the sample and the crystal, which is critical for consistent path length in ATR measurements.

- Instrument Parameters:
- Spectral Range: 4000–400 cm^−1^.
- Spectral Resolution: 4 cm^−1^.
- Number of Scans: 32 scans per spectrum.
- Scanner Velocity: 0.6329 cm/s.
- Apodization Function: Happ-Genzel.
- Detector: DTGS (Deuterated Triglycine Sulfate).
- Beam Splitter: KBr.

Each sample was measured three times (reloading the sample for each measurement to account for sampling heterogeneity). The three spectra were averaged to produce a single representative spectrum for each sample. A total of 7468 wavelength points were recorded for each sample. The crystal was cleaned with 70% ethanol and dried with lens tissue between samples to prevent cross-contamination.

### 3.5. Sample Set Partitioning

Appropriate sample partitioning is critical for developing robust prediction models, particularly in small-sample scenarios where the representativeness of calibration and prediction sets directly affects model generalization. To identify the optimal partitioning strategy, two widely used algorithms were systematically compared: the sample set partitioning based on joint X-Y distances (SPXY) and the Kennard-Stone (KS) algorithm.

The SPXY algorithm considers both spectral variables (X) and reference values (Y) when calculating sample distances, ensuring that both sets adequately represent the chemical and spectral variability of the entire sample population [30]. This joint consideration is particularly advantageous for quantitative spectroscopic analysis, where the relationship between spectral features and target properties is the primary modeling objective. In contrast, the KS algorithm selects samples based solely on Euclidean distances in the spectral space, ensuring uniform spatial coverage but potentially neglecting the distribution of target values.

(3) $$d_{xy}\left(p,q\right)=\frac{d_{x}\left(p,q\right)}{\underset{p,q}{\max}d_{x}\left(p,q\right)}+\frac{d_{y}\left(p,q\right)}{\underset{p,q}{\max}d_{y}\left(p,q\right)}$$

The comparative evaluation revealed (Table 7) that SPXY demonstrated superior performance with a 4.7% improvement in RPD (2.258 vs. 2.158) and excellent reproducibility (RPD standard deviation = 0.000). The advantage of SPXY can be attributed to its joint consideration of both spectral and chemical information, which ensures that the calibration set contains samples spanning the full range of polyphenol concentrations while maintaining spectral diversity. This is particularly important for *Lonicera caerulea* samples collected from different geographical regions, where both spectral characteristics and polyphenol content may vary systematically. Based on these results, SPXY was selected to divide the 191 samples into a calibration set (*n* = 152, 80%) and a prediction set (*n* = 39, 20%).

### 3.6. Spectral Preprocessing

Spectral preprocessing is essential for enhancing signal quality and improving chemical interpretability in spectroscopic modeling. Raw MIR spectra of powdered samples are typically affected by baseline drift, light scattering due to particle size variations, and overlapping absorption bands, all of which can obscure the chemical information relevant to polyphenol content. To identify the optimal preprocessing strategy, ten methods representing different correction approaches were systematically evaluated:

- No preprocessing (raw spectra)—baseline reference
- Savitzky–Golay (SG) smoothing (window = 11, polynomial order = 2)—noise reduction
- SG first derivative (window = 11, polynomial order = 2)—baseline correction and peak enhancement
- Standard normal variate (SNV)—scatter correction through row-wise normalization
- SNV + SG smoothing—combined scatter correction and noise reduction
- SNV + SG first derivative—combined scatter correction and derivative enhancement
- Multiplicative scatter correction (MSC)—scatter correction using mean spectrum reference
- MSC + SG smoothing—combined MSC and noise reduction
- MSC + SG first derivative—combined MSC and derivative enhancement
- Detrending—polynomial baseline removal

The first derivative was preferred over the second derivative because it provides sufficient baseline correction and peak resolution enhancement while preserving more spectral information and introducing less noise amplification, which is particularly important for the relatively broad absorption bands characteristic of polyphenolic compounds in the MIR region.

Each preprocessing method was evaluated using PLS regression with 5-fold cross-validation on the calibration set. The coefficient of determination (*R*^2^) and ratio of performance to deviation (RPD) on the prediction set were used as selection criteria, with the method achieving the highest values selected for all subsequent analyses to ensure consistency throughout the modeling workflow.

### 3.7. Variable Selection Methods

Variable selection is crucial for addressing the curse of dimensionality in high-dimensional small-sample spectroscopic data. With 7468 wavelengths and only 191 samples (*p* >> *n*), direct modeling without variable selection would likely result in severe overfitting and poor generalization. Moreover, many spectral variables contain redundant information or noise that does not contribute to polyphenol prediction. To develop a robust and interpretable model, we proposed a hybrid variable selection strategy that combines linear and nonlinear feature evaluation methods, and compared it with the classical CARS approach.

#### 3.7.1. PLS-VIP Selection

The variable importance in projection (VIP) score was calculated from a PLS model to identify wavelengths with significant linear contributions to polyphenol prediction. PLS-VIP was chosen because it directly quantifies each variable’s contribution to the explained variance in the target property while accounting for the multicollinearity inherent in spectral data. The VIP score for the *j*-th variable is calculated as:

(4) $$VIP_{j}=\sqrt{\frac{p\times \sum_{a=1}^{A}\left(SS_{a}\times {\left(w_{aj}/\|w_{a}\|\right)}^{2}\right)}{\sum_{a=1}^{A}SS_{a}}}$$

where p is the number of variables, A is the number of latent variables, SS_a is the sum of squares explained by the a-th component, and w_{a,j} is the weight of the j-th variable for the a-th component. Variables with VIP ≥ 1.0 were considered important for the linear model, as this threshold indicates above-average contribution to the model [30]. This criterion ensures that selected wavelengths have demonstrable chemical relevance to polyphenol content from a linear modeling perspective.

#### 3.7.2. Random Forest Regression Importance

While PLS-VIP captures linear relationships, the relationship between spectral features and polyphenol content may also involve nonlinear patterns and complex interactions.RFR was therefore employed to evaluate variable importance from a nonlinear perspective. RFR was chosen over other nonlinear methods (e.g., Lasso, elastic net) because it naturally handles high-dimensional data, captures variable interactions, and provides stable importance rankings through ensemble averaging. The importance of each variable was calculated based on the mean decrease in impurity (Gini importance) across all trees in the forest. The top 30% of variables ranked by normalized importance were selected as nonlinearly important variables. This threshold was determined through preliminary experiments balancing dimensionality reduction with information retention.

#### 3.7.3. Construction and Optimization of Hybrid Variable Selection Strategies

To identify the optimal feature subset that balances dimensionality reduction with information retention, a systematic screening process was conducted. Instead of arbitrarily selecting a single threshold, we constructed a candidate pool consisting of five distinct selection strategies based on different stringency levels of PLS-VIP and RFR. The candidate strategies were designed as follows:

Single-Criterion Strategies: Relaxed Linear Selection (PLS-VIP-0.8): Variables with VIP ≥ 0.8 were retained to preserve maximum information. Strict Linear Selection (PLS-VIP-1.2): Variables with VIP ≥ 1.2 were retained to highlight highly significant linear features. Strict Nonlinear Selection (RFR-top10%): Only the top 10% most important variables from RFR were retained to capture the strongest nonlinear features.

Hybrid Intersection Strategies: To combine the complementary strengths of linear and nonlinear evaluations, two intersection-based schemes were designed: High-Stringency Hybrid (VIP-1.2∩RFR-20%): Intersection of VIP ≥ 1.2 and the top 20% RFR variables, aiming for a compact, high-confidence subset. Balanced Hybrid (VIP-1.0∩RFR-30%): Intersection of VIP ≥ 1.0 (standard threshold) and the top 30% RFR variables, aiming to balance feature stability and spectral coverage.

Screening Protocol: All five candidate strategies were subjected to a rigorous evaluation using the XGBoost model with fixed hyperparameters (details in Section 3.9, Stage 1). Based on the comparative results (presented in Section 2.2.4), the Balanced Hybrid strategy (VIP1.0∩RFR30%) demonstrated the highest prediction accuracy and stability. Therefore, it was defined as the optimal strategy for this study.

The intersection logic of the optimal strategy is defined as:

(5) $$S_{hybrid}=\{v_{i}|VIP_{i}\geq 1.0\}\cap \{v_{i}|RFR_{I}mp_{i}\in Top\;30\%\}$$

This intersection-based design ensures that selected variables are validated from both perspectives. The rationale is threefold:

- Variables important only in linear models may lack robustness for nonlinear modeling;
- Variables important only in nonlinear models may lack clear chemical interpretability;
- The intersection naturally achieves substantial dimensionality reduction while retaining wavelengths with both chemical relevance (PLS-VIP) and statistical stability under nonlinear modeling (RFR). This hybrid design is particularly suited for small-sample scenarios where single-criterion selection may be unstable.

#### 3.7.4. CARS Method (Comparative Baseline)

**Figure 7:**
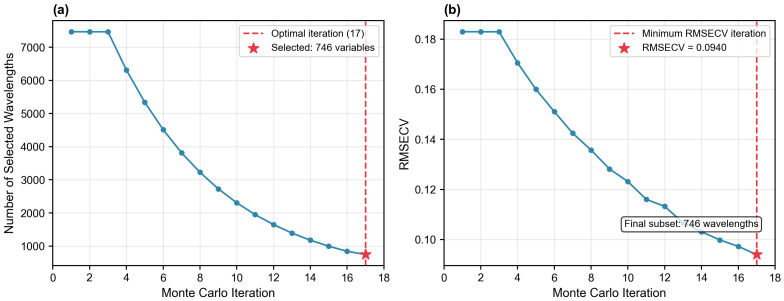
CARS variable selection process. (**a**) Evolution of the number of selected wavelengths across 18 Monte Carlo iterations; (**b**) RMSECV values as a function of iterations. The red star and dashed line indicate the optimal iteration (17) achieving the minimum RMSECV (0.0940) with a final subset of 746 variables.

To benchmark the proposed hybrid strategy, competitive adaptive reweighted sampling (CARS) was implemented as a comparative baseline. CARS is a widely used variable selection method in chemometrics that employs Monte Carlo sampling combined with an exponentially decreasing function and adaptive reweighted sampling to iteratively select optimal variables. In this study, the algorithm was configured with 50 iterations and 10 Monte Carlo runs. The variable subset corresponding to the minimum root mean square error of cross-validation (RMSECV) was selected as the final feature set (Figure 7). CARS was chosen as the baseline because it represents a fundamentally different selection philosophy—iterative optimization based on model performance rather than explicit importance scoring—providing a robust reference for evaluating the effectiveness of the hybrid strategy.

### 3.8. Machine Learning Models

To verify that the selected variables contain robust information for polyphenol prediction regardless of the specific modeling algorithm, four machine learning models spanning different algorithmic paradigms were employed for multi-model validation:

- Partial Least Squares Regression (PLS): A classical linear chemometric method that projects both X and Y onto latent variables to maximize covariance. PLS was included as the baseline linear model due to its widespread use in spectroscopic analysis and its ability to handle multicollinearity.
- Random Forest Regression (RFR): An ensemble method that constructs multiple decision trees using bootstrap sampling and averages their predictions. RFR was included to represent bagging-based ensemble methods and to assess nonlinear modeling capability.
- Support Vector Regression (SVR): A kernel-based method that maps data to a high-dimensional feature space for nonlinear regression. SVR with RBF kernel was included to represent kernel-based approaches that excel in small-sample scenarios.
- Extreme Gradient Boosting (XGBoost): A gradient boosting algorithm that sequentially builds decision trees to minimize prediction errors with built-in regularization. XGBoost was included as the state-of-the-art boosting method known for its strong performance in structured data problems.

The diversity of these models—covering linear, bagging, kernel-based, and boosting approaches—ensures that the validation results reflect the intrinsic quality of the selected variables rather than the suitability of any particular algorithm.

### 3.9. Three-Stage Hyperparameter Tuning Strategy

A critical challenge in high-dimensional small-sample modeling is the risk of overfitting during hyperparameter optimization. With only 152 training samples and potentially thousands of variables, aggressive hyperparameter tuning on the same dataset can lead to selecting parameter combinations that are overfitted to the specific sample set rather than generalizable to new data. To balance model fairness, computational efficiency, and overfitting control, a three-stage hyperparameter tuning strategy was implemented, with each stage serving a distinct purpose:

#### 3.9.1. Variable Selection Comparison (Fixed Parameters)

To ensure fair comparison among different variable selection schemes without parameter-induced bias, all schemes were evaluated using XGBoost with identical fixed parameters (max_depth = 3, learning_rate = 0.1, n_estimators = 300). This design isolates the effect of variable selection from hyperparameter optimization, allowing direct assessment of each scheme’s intrinsic quality.

#### 3.9.2. Multi-Model Validation (Light Tuning)

As shown in Table 8,after identifying the optimal variable subset (VIP1.0∩RFR30%), its universality was validated across four models with equal-budget light hyperparameter tuning. The key principle at this stage is to provide each model with a fair opportunity to perform well without aggressive optimization that could mask the true quality of the selected variables:

**Table 8:** Hyperparameter search strategy for model optimization.

Model	Parameter Range	Search Strategy
PLS	n_components: 5–15	Grid search
RFR	max_depth: {4, 6, 8, 10}, min_samples_leaf: {2, 3, 5}	Small grid search
SVR	$C:\{1,10,100\},\mathrm{gamma}:\{1\times 10^{-3}$$,1\times 10^{-2}$$,1\times 10^{-1}$}	3 × 3 log-grid search
XGBoost	max_depth: {3, 4, 5}, learning_rate: {0.05, 0.1}	Small random search

All models were evaluated using the same 5-fold cross-validation splits to ensure comparable and reproducible results. This stage answers the question: “Given reasonable tuning, how does the VIP1.0∩RFR30% variable subset perform across different modeling paradigms?”.

#### 3.9.3. Final Model Optimization (Fine Tuning)

Only after confirming the universality of the selected variables was the best-performing model (XGBoost) subjected to fine hyperparameter optimization (Table 9). This sequential approach ensures that intensive optimization is applied only to the final model configuration, minimizing the risk of overfitting to the specific dataset:

All hyperparameter searches were conducted using cross-validation on the calibration set only. Critically, the independent prediction set was reserved exclusively for final evaluation and was used only once after all model development was complete. This strict separation prevents information leakage and ensures that the reported prediction performance reflects true generalization capability rather than optimistic estimates from repeated testing.

### 3.10. Model Evaluation Metrics

Model performance was evaluated using the following metrics:

Coefficient of Determination (*R*^2^): Measures the proportion of variance explained by the model.

(6) $$R^{2}=1-\frac{\sum_{i=1}^{n}(y_{i}-{\hat{y}}_{i}{)}^{2}}{\sum_{i=1}^{n}(y_{i}-\overset{-}{y}{)}^{2}}$$

Root Mean Square Error (RMSE): Measures the average prediction error.

(7) $$RMSE=\sqrt{\frac{1}{n}\sum_{i=1}^{n}(y_{i}-{\hat{y}}_{i}{)}^{2}}$$

Ratio of Performance to Deviation (RPD): Ratio of the standard deviation of reference values to RMSE.

(8) $$RPD=\frac{SD_{ref}}{RMSEP}=\frac{\sqrt{\frac{1}{n-1}\sum_{i=1}^{n}(y_{i}-\overset{-}{y}{)}^{2}}}{RMSEP}$$

RPD values are interpreted as: RPD < 1.5 (unreliable), 1.5 ≤ RPD < 2.0 (rough screening), 2.0 ≤ RPD < 2.5 (approximate quantification), 2.5 ≤ RPD < 3.0 (good prediction), RPD ≥ 3.0 (excellent prediction) [22].

## 4. Conclusions

This study developed a quantitative prediction model for polyphenol content in *Lonicera caerulea* using mid-infrared spectroscopy combined with a novel hybrid variable selection strategy specifically designed for high-dimensional small-sample scenarios. The main conclusions are:

Optimal preprocessing: MSC combined with SG first derivative was identified as the optimal preprocessing method for MIR spectra of *Lonicera caerulea*, achieving *R*^2^ = 0.8735 and RPD = 2.8121 with PLS regression. The synergistic effect of scatter correction and derivative enhancement effectively preserved chemical information while reducing physical interference.

Effective dimensionality reduction: The proposed hybrid variable selection strategy (VIP1.0∩RFR30%) effectively reduced dimensionality by 86.8% (from 7468 to 984 wavelengths) while maintaining excellent prediction performance, demonstrating that the intersection of linear and nonlinear importance criteria provides robust variable selection.

Superior performance over classical methods: The VIP1.0∩RFR30% strategy significantly outperformed the classical CARS method, with improvements of 16.3% in *R*^2^ and 55.2% in RPD, highlighting the limitations of purely iterative optimization-based methods in high-dimensional small-sample scenarios.

Cross-model universality: The selected 984 wavelengths demonstrated universality across four machine learning models representing different algorithmic paradigms (PLS, RFR, SVR, XGBoost), with all models achieving RPD > 2.5, confirming that the selected variables contain intrinsically robust spectral–chemical information.

Excellent prediction performance: The optimized XGBoost model achieved excellent prediction performance with *R*^2^ = 0.92, RMSE = 0.098, and RPD = 3.47 on the independent test set, meeting the criteria for “excellent prediction capability” suitable for quality control applications.

Effective overfitting control: The three-stage hyperparameter tuning strategy effectively prevented overfitting in the high-dimensional small-sample scenario, as evidenced by the small gap between calibration and prediction performance.

Chemical interpretability: The selected wavelengths showed clear chemical relevance to polyphenolic functional groups (phenolic hydroxyl, aromatic rings, carbonyl groups), enhancing model interpretability and providing mechanistic insights into the spectral basis of polyphenol quantification.

These findings demonstrate the feasibility of MIR-based modeling for non-destructive polyphenol assessment in berries and establish a methodological foundation for industrial applications and standardization of *Lonicera caerulea* quality control. The proposed hybrid variable selection strategy and three-stage tuning approach can be extended to other high-dimensional small-sample spectroscopic applications in food analysis where robust prediction and chemical interpretability are required.

## Figures and Tables

**Table 1:** Performance comparison of different spectral preprocessing methods using PLS regression.

Rank	Preprocessing Method	Test *R*^2^	RMSE	RPD
1	MSC + SG first derivative	0.8735	0.1206	2.8121
2	SNV + SG first derivative	0.8735	0.1206	2.8120
3	SG first deriv-ative	0.8661	0.1241	2.7324
4	SNV	0.8299	0.1399	2.4245
5	SNV + SG smoothing	0.8285	0.1405	2.4148
6	MSC	0.8224	0.1429	2.3732
7	MSC + SG smoothing	0.8208	0.1436	2.3624
8	SG smoothing	0.8080	0.1486	2.2820
9	No prepro-cessing	0.8061	0.1494	2.2708
10	Detrending	0.7616	0.1656	2.0479

**Table 2:** Performance comparison of different variable selection strategies using XGBoost.

Rank	Selection Strategy	Variables	Test *R*^2^	RPD
1	VIP1.0∩RFR30%	984	0.9097	3.3270
2	RFR-top10%	746	0.8986	3.1407
3	VIP-1.2∩RFR-20%	604	0.8928	3.0546
4	PLS-VIP-1.2	1690	0.8799	2.8850
5	PLS-VIP-0.8	4057	0.8496	2.5788
6	CARS	746	0.7823	2.1431

**Table 3:** Performance of different models using VIP1.0∩RFR30% variables (984 wavelengths).

Model	*R*^2^_cv	RMSECV	RPD_cv	Test *R*^2^	Test RMSE	Test RPD
PLS	0.7337	0.1582	1.9390	0.8427	0.1312	2.5211
RFR	0.8215	0.1324	2.3742	0.8625	0.1235	2.6971
SVR	0.8032	0.1378	2.2589	0.8532	0.1268	2.6245
XGBoost	0.8427	0.1268	2.5211	0.8826	0.1162	2.9190

**Table 4:** Performance of the optimized XGBoost model.

Dataset	*R* ^2^	RMSE	RPD
Calibration (CV)	0.9633	0.0632	—
Prediction	0.9168	0.0978	3.4674

**Table 5:** Chemical interpretation of key spectral regions in the selected wavelengths.

Dataset	*R* ^2^	RMSE
Calibration (CV)	0.9633	0.0632
Prediction	0.9168	0.0978

**Table 6:** Comparison with previous spectroscopic studies on berry polyphenol quantification.

Study	Sample	Spectroscopy	Samples	Variables	Model	*R* ^2^	RPD
This study	*L. caerulea*	MIR	191	984 (VIP∩RFR)	XGBoost	0.9168	3.4674
[27]	Blueberry	NIR		Full spectrum	PLS	0.85	2.58
[28]	Grape	MIR		CARS	PLS	0.88	2.89
[29]	Cranberry	NIR		SPA	SVR	0.82	2.36

**Table 7:** Comparison of Sample Set Partitioning Methods.

Partitioning Method	Test *R*^2^	RMSE	RPD	RPD Std
SPXY	0.8735	0.1206	2.258	0.000
Kennard-Stone	0.8612	0.1264	2.158	0.012

**Table 9:** XGBoost hyperparameter ranges and descriptions.

Parameter	Search Range	Description
n_estimators	50–500	With early stopping
max_depth	2–6	Tree depth control
learning_rate	0.01–0.3	Learning rate
subsample	0.6–1.0	Sample ratio
colsample_bytree	0.6–1.0	Feature ratio
reg_alpha	0–1.0	L1 regularization
reg_lambda	0–2.0	L2 regularization

## Data Availability

The data presented in this study are available on request from the corresponding author.

## Abbreviations

The following abbreviations are used in this manuscript:

ATR-FTIR	Attenuated Total Reflectance-Fourier Transform Infrared Spectroscopy
CARS	Competitive Adaptive Reweighted Sampling
DW	Dry Weight
GAE	Gallic Acid Equivalents
KS	Kennard-Stone
MIR	Mid-Infrared
MSC	Multiplicative Scatter Correction
NIR	Near-Infrared
PLS	Partial Least Squares
RFR	Random Forest Regression
RMSE	Root Mean Square Error
RMSEC	Root Mean Square Error of Calibration
RMSECV	Root Mean Square Error of Cross-Validation
RMSEP	Root Mean Square Error of Prediction
RPD	Ratio of Performance to Deviation
SG	Savitzky–Golay
SNV	Standard Normal Variate
SPXY	Sample set Partitioning based on joint X-Y distances
SVR	Support Vector Regression
TPC	Total Polyphenol Content
UV	Ultraviolet
VIP	Variable Importance in Projection
